# Zeolite NaP1 Functionalization for the Sorption of Metal Complexes with Biodegradable *N*-(1,2-dicarboxyethyl)-D,L-aspartic Acid

**DOI:** 10.3390/ma14102518

**Published:** 2021-05-12

**Authors:** Dorota Kołodyńska, Yongming Ju, Małgorzata Franus, Wojciech Franus

**Affiliations:** 1 Department of Inorganic Chemistry, Faculty of Chemistry, Institute of Chemical Sciences, Maria Curie-Skłodowska University, Maria Curie Skłodowska Square 2, 20-031 Lublin, Poland; 2 Nanjing Institute of Environmental Sciences, Ministry of Ecology and Environment (MEE), Nanjing 210042, China; juyongming@scies.org; 3 Innovative Laboratory for Environmental Functional Materials and Environmental Applications of Microwave Irradiation, South China Sub-Center of the State Environmental Dioxin Monitoring Center, South China Institute of Environmental Sciences, Ministry of Environmental Protection, Guangzhou 510655, China; 4 Department of Construction, Faculty of Civil Engineering and Architecture, Lublin University of Technology, Nadbystrzycka 40, 20-618 Lublin, Poland; m.franus@pollub.pl; 5 Department of Geotechnics, Civil Engineering and Architecture Faculty, Lublin University of Technology, Nadbystrzycka 40, 20-618 Lublin, Poland; w.franus@pollub.pl

**Keywords:** fly ash, zeolite, chitosan, biodegradable complexing agents

## Abstract

The possibility of application of chitosan-modified zeolite as sorbent for Cu(II), Zn(II), Mn(II), and Fe(III) ions and their mixtures in the presence of *N*-(1,2-dicarboxyethyl)-D,L-aspartic acid, IDHA) under different experimental conditions were investigated. Chitosan-modified zeolite belongs to the group of biodegradable complexing agents used in fertilizer production. NaP1CS as a carrier forms a barrier to the spontaneous release of the fertilizer into soil. The obtained materials were characterized by Fourier transform infrared spectroscopy (FTIR); surface area determination (ASAP); scanning electron microscopy (SEM-EDS); X-ray fluorescence (XRF); X-ray diffraction (XRD); and carbon, hydrogen, and nitrogen (CHN), as well as thermogravimetric (TGA) methods. The concentrations of Cu(II), Zn(II), Mn(II), and Fe(III) complexes with IDHA varied from 5–20 mg/dm^3^ for Cu(II), 10–40 mg/dm^3^ for Fe(III), 20–80 mg/dm^3^ for Mn(II), and 10–40 mg/dm^3^ for Zn(II), respectively; pH value (3–6), time (1–120 min), and temperature (293–333 K) on the sorption efficiency were tested. The Langmuir, Freundlich, Dubinin–Radushkevich, and Temkin adsorption models were applied to describe experimental data. The pH 5 proved to be appropriate for adsorption. The pseudo-second order and Langmuir models were consistent with the experimental data. The thermodynamic parameters indicate that adsorption is spontaneous and endothermic. The highest desorption percentage was achieved using the HCl solution, therefore, proving that method can be used to design slow-release fertilizers.

## 1. Introduction

In the groups of different low-cost substances used for fertilization, fly ashes, slag materials, zeolites and their modified forms—organozeolitic biofertilizers, surfactant modified zeolites (especially by the hexadecyltrimethylammonium (HDTMA)) or nanozeolites, as well as different types of biochars, etc., should be mentioned [1,2,3,4,5,6,7,8,9]. They have influenced soil properties and crop productivity [10]. In particular, zeolites—microporous crystalline aluminosilicates with structures formed by tetrahedrons of [SiO_4_]^4−^ and [AlO_4_]^5−^ linked through oxygen atoms—added to the soil can increase agricultural crop growth by as much as 50%, regulate humidity in soil in long period of drought, prevent diseases of plant roots, adsorb fertilizers and regulate their uptake, and, at the same time, reduce the adsorption of toxic ions by plants and promote nitrification of nitrogen [1,11]. They can also be used as pesticides, insecticides, antibacterial agents, and growth simulator carriers. In the group of enhanced efficiency fertilizers, special attention is paid to slow- and controlled-release nitrogen/phosphorous and stabilized nitrogen/phosphorous fertilizers [12,13]. These special kinds of new fertilizers, rich in nitrogen and phosphorus, can be prepared by modification of zeolites by Al(III), Fe(II), or Mn(II) ions and incorporation of ammonium and phosphates from wastewaters. The growing requirements in the use of mineral fertilizers determine further development in this field, especially with a better biodegradable profile. Also, those with metal ions in the chelate forms are of significant importance because micronutrients delivered to plants undergo various soil chemical transformations that significantly reduce their availability. According to regulation (EC) No 2003/2003 of the European Parliament and of the Council of 13 October 2003 (as amended) relating to fertilizer, so far, only a few substances have been used to this aim. The most important, namely, ethylenediaminetetracetic acid (EDTA), ethylenediamine-*N*-*N*′bis(o-hydroxyphenylacetic) acid (o,oEDDHA), *N*-(1,2-dicarboxyethyl)-D,L-aspartic acid (IDHA), and ethylenediaminedisuccinic acid (EDDS), as well as (*N*,*N*′-bis(2-hydroxy-5-methylbenzyl)ethylenediamine-*N*,*N*′-diacetic acid) (HJB) and (*N*,*N*′-bis(2- hydroxybenzyl)ethylenediamine-*N*,*N*’-diacetic acid) (HBED), have been applied. Moreover, additional factors for crop yield, such as soil type, climatic factors, and type of plant should be taken into account. For such different conditions, fly ashes and zeolites are recommended due to numerous advantages [14]. In this paper, an approach to examine Cu(II), Zn(II), Mn(II), and Fe(III) complexes with the presence of biodegradable IDHA sorption using chitosan-modified zeolite (NaP1CS) was employed. The originality of the paper lies in the opportunity to characterize physicochemical and adsorptive properties of NaP1CS. These collective actions make it easier to bring together scientific and practical knowledge to the future application of NAP1CS for slow-release fertilizers. In this context, the studies contribute to the development of practical new strategies, tools, and incentives for the application of low-cost materials (fly ashes, zeolites, and chitosan) for agriculture. To examine NaP1CS’ applicability, the effects of parameters such as the concentration of Cu(II), Zn(II), Mn(II), and Fe(III) complexes with IDHA, pH value, time, and temperature on sorption, as well as desorption efficiency were tested. The Langmuir, Freundlich, and Temkin adsorption models were applied to describe the experimental data.

## 2. Materials and Methods

### 2.1. Materials

The fly ash (FA) used for the synthesis of zeolite NaP1 was supplied by the electric power plant (Kozienice, Poland). Production of zeolite NaP1 was performed based on the hydrothermal synthesis of FA with NaOH at atmospheric pressure on a pilot-scale (24 h at 353 K), as was previously described [15]. According to [16], 20 kg of FA, 12 kg of NaOH, and 90 dm^3^ of H_2_O can be used to obtain NaP1. Chitosan flakes (CS) with the deacetylation degree > 75% (Sigma Aldrich, Warsaw, Poland) were dissolved in 1% solution of glycolic acid and mixed using a magnetic stirrer at 1000 rpm at 294 K for 24 h, which significantly contributed to reduction of solution viscosity. Next, zeolite was added to the chitosan solution. The mass ratio of zeolite to chitosan was, 8:1. Afterwards the resulting product was precipitated by 1 M NaOH solution and then filtered and washed with distilled water to neutral pH, dried, and ground as the last step.

The stock solutions of Cu(II), Zn(II), Mn(II), and Fe(III) ions of the concentration 1 g/dm^3^ were prepared by dissolving proper amounts of CuCl_2_·2H_2_O, ZnCl_2_, MnCl_2_·4H_2_O, and FeCl_3_·6H_2_O (Avantor Performance Materials) and IDHA in distilled water. Iminodisuccinic acid (IDHA), known as Baypure CX 100 (Lanxess), is an innovative new generation biodegradable complexing agent characterized by a good eco-toxicological profile. It protects metal ions against precipitation up to a pH of 6.0. The stability constant of IDHA is moderate. IDHA is also friendly for the environment; it is fully biodegradable according to the OECD regulations (OECD 301E 78% after 28 days and OECD 302B 89% after 28 days). Appropriate concentrations were obtained by diluting the stock solutions.

### 2.2. Instruments

To identify the functional groups present on the adsorbent surface, the Fourier transform infrared spectroscopy (FTIR) method was used. The FTIR spectra were measured in the wavenumber ranging from 4000 to 400 cm^−1^ by the Cary 630 FTIR spectrometer (Agilent Technologies, Santa Clara, CA, USA).

Carbon, hydrogen, and nitrogen (CHN) analyses were also made using the elemental analyzer CHN 2400 (Perkin-Elmer, Waltham, MA, USA).

To characterize the adsorbent before and after adsorption, the combination of scanning electron microscopy and energy dispersive X-ray spectroscopy (SEM-EDS) using LEO 1430 VP microscope and Quantax 200 with the detector XFlash 4010 (Bruker AXS, Ettlingen, Baden-Württemberg, Germany) spectrometer was also applied.

To obtain textural parameters of the composite before and after modification, as well as after the adsorption process, the measurements of N_2_ adsorption/desorption isotherms at 77 K were conducted. To this aim, the samples were analyzed using ASAP 2420 (Micromeritics, Inc., Norcross, GA, USA). 

Analysis of chemical composition of zeolites and fly ashes was conducted using semi-quantitative energy dispersive X-ray fluorescence (ED-XRF) method on an Epsilon 3 (Panalytical, Eindhoven, The Netherlands) apparatus. The samples were used in their original powdered form in the amount of 3 g per sample.

The mineral/phase composition of zeolites was determined using the X-ray diffraction powder method (XRD) using an X’pert MPD X-ray diffractometer (Panalytical, Eindhoven, The Netherlands) with a goniometer PW 3020 and X-ray source anode Cu (K_α_) and a graphite monochromator. Diffraction patterns were recorded, and HighScore Pro software (version 3.0, Eindhoven, The Netherlands) was used to process diffraction data. The identification of mineral phases was based on the PCPDFWIN ver. 1.30 formalized by JCPDS-ICDD. Before measurement, the samples were milled and sieved.

Thermal behavior of composite was determined by thermogravimetric analysis using Jupiter STA 449F3 analyzer (Netzsch GmBH, Selb, Germany). A sample of 10 mgwas tested under a nitrogen atmosphere and heated in a temperature range of 30–1000 °C with a 10 °C/min step.

### 2.3. Methods—Kinetic and Adsorption Experiments

Kinetic and adsorption experiments were performed by the static method; 0.1 g of NaP1CS samples were mixed with 20 cm^3^ solutions containing Cu(II), Fe(III), Mn(II), and Zn(II) complexes with IDHA. Analogous studies were carried out for NaP1. In the case of four-component solutions, the Cu(II), Fe(III), Mn(II), and Zn(II) ratio was 1:2:4:2, which approximately corresponds to typical commercial fertilizers. Kinetic experiments were performed at four different concentrations. Therefore, the initial concentrations of Cu(II), Fe(III), Mn(II), and Zn(II) complexes with IDHA were varied from 5–20 mg/dm^3^ for Cu(II), 10–40 mg/dm^3^ for Fe(III), 20–80 mg/dm^3^ for Mn(II), and 10–40 mg/dm^3^ for Zn(II), respectively. The adsorbents were mixed with the solution for 1–120 min (shaking amplitude 7, temperature 293 K). To calculate the kinetic parameters, different models were proposed: pseudo-first order, pseudo-second order, and intraparticle diffusion. Following that, 1 M NaOH and 1 M HCl were applied to adjust the initial pH of the solution, respectively. Desorption experiments were performed in the 0.5 M NaCl, NaOH, KCl, CH_3_OH, and HCl solutions, respectively. Then, 20 cm^3^ of solution was contacted with 0.1 g of adsorbent after the adsorption equilibrium of Cu(II), Fe(III), Mn(II), and Zn(II) complexes with IDHA. Desorption was conducted at room temperature with the shaking amplitude 7 for 120 min. The samples after the adsorption or desorption process were filtered, and the concentration of Cu(II), Fe(III), Mn(II), and Zn(II) was determined using the inductively coupled plasma optical emission spectrometry (ICP-OES) and spectrometer ICP-OES 720 ES (Varian, Melbourne, Australia). The ICP-OES instrument was calibrated using the appropriate standards. For the preparation of all standards and blank samples, the ultrapure nitric acid was used to avoid any matrix interference. The relative standard deviation (RSD) for the triplicate analysis was within 5%.

### 2.4. Calculations

To calculate the kinetic parameters, different models were proposed: pseudo-first order, pseudo-second order, and intraparticle diffusion [17]. The pseudo-first order equation is presented as follows:

ln(q_e_ − q_t_) = lnq_e_ − k_1_t or q_t_ = q_e_[1 − exp(−k_1_t)], (1)

where k_1_ is the rate constant of the pseudo first order model (1/min) and q_e_ and q_t_ are the adsorption capacities (mg/g) at equilibrium and at time t (min), respectively. The adsorption capacity at equilibrium q_e_ was determined from an experiment using the fractional uptake F equal calculated as F = q_t_/q_e_.

The pseudo second order equation is presented in the equation below:

t/q_t_ = t/q_e_ + 1/k_2_q_e_^2^ or q_t_ = k_2_q_e_^2^t/1 + k_2_q_e_t, (2)

where k_2_ is the rate constant of the pseudo second order model (g/mg min).

Furthermore, the diffusion model given by the Weber−Morris equation was also considered:

q_t_ = k_i_t^1/2^ + C, (3)

where q_t_ is the adsorption capacity (mg/g) at time t, t is the contact time (min), and k_i_ (mg/g min^1/2^) and C (mg/g) are the Weber−Morris diffusion constants.

The linear form of the Langmuir isotherm is presented in Equation (4):

C_e_/q_e_ = C_e_/q_m_ + 1/(q_m_K_L_), (4)

where q_e_ is defined as previously, C_e_ is the equilibrium concentration of complex in solution (mg/dm^3^), q_m_ is the monolayer adsorption capacity of the adsorbent (mg/g), and K_L_ is the Langmuir constant (dm^3^/mg), which represents the adsorption free energy.

The adsorption character can be determined by means of the R_L_ parameter obtained from the Langmuir model. In contrast to the Langmuir isotherm, the Freundlich one assumes a multilayer adsorption. The linear form of the Freundlich isotherm is as follows:

logq_e_ = logK_F_ + (1/n)logC_e_, (5)

The values of 1/n and K_F_ were designated from the slope and intercept of the Freundlich plots (logq_e_ vs. logC_e_). K_F_ and n are the Freundlich constants, K_F_ is related to the adsorption capacity (mg/g), and 1/n is an empirical parameter related to the intensity of the reaction and energy heterogeneity [18].

Another model is the Temkin isotherm, which assumes that the heat of adsorption decreases linearly with the increase in coverage of adsorbent. The linear form of the Temkin isotherm is expressed by Equation (6).

q_e_ = BlnA + BlnC_e_, (6)

where B = (RT/b) (b is the Temkin constant related to heat of adsorption) and A is the equilibrium binding constant, R is the gas constant (8.314 J/mol K), and T is temperature (K) [19]. The values of B and A were obtained by plotting q_e_ vs. lnC_e_ and calculated from the slope and intercept, respectively.

The Dubinin–Radushkevich (D–R) isotherm model explains the adsorption on both homogenous and heterogeneous surfaces. The linear form of the D–R isotherm is given by Equation (7):

lnq_e_ = lnX_m_ − βε^2^, (7)

where X_m_ is the maximum adsorption capacity (mg/g) and ε is a constant associated with the adsorption energy (mol^2^/kJ^2^). ε is the Polanyi potential and can be calculated as follows:

ε = RTln(1 + 1/C_e_), (8)

where R is the gas constant (8.314 J/mol K), T is the temperature (K), and C_e_ is the equilibrium concentration of the adsorbate (mg/L).

Gibb’s free energy (ΔG°) was determined from Equation (9):

ΔG° = RTln(K_c_), (9)

where R is the gas constant (8.314 J/mol K), T is the temperature (K), and K_c_ is the equilibrium constant. (ΔH°) and (ΔS°) were calculated from the slope and intercept of the van’t Hoff plots of lnK_c_ vs. 1/T.

## 3. Results

### 3.1. NaP1CS Preparation and Characterization

Zeolite NaP1 can be easily synthesized from various wastes, such as fly ashes, by hydrothermal treatment. The analogous procedure was proposed in [20] for slow release of fertilizer in the ammonium form based on NaP1 zeolite and hydroxyapatite. In our paper, FA was used as the initial material. According to the ASTM D 3987-85 2001 test for FA analysis and considering conditions such as eluting agent H_2_O, solution: FA ratio equal to 20:1, elution time 6 h, and temperature 293–298 K, it was proven that FA is safe and stable. The composition of the FA water eluate is as follows: Fe(II,III) 0.024, Mn(II) 0.005, Ca(II) 591, Mg(II) 0.020, Na(I) 28.62, K(I) 18.41, Zn(II) 0.007, Cd(II) < 0.001, Cu(II) 0.004, Pb(II) 0.004, Ni(II) 0.002, Sr(II) 2.07, Cr(III,VI) 0.56, Cr(VI) 0.49, Cr(III) 0.07, B(III) 1.15 mg/dm^3^. The obtained results also show that FA consists mainly of mullite and quartz. The main constituents are presented in Table 1.

**Figure 1:**
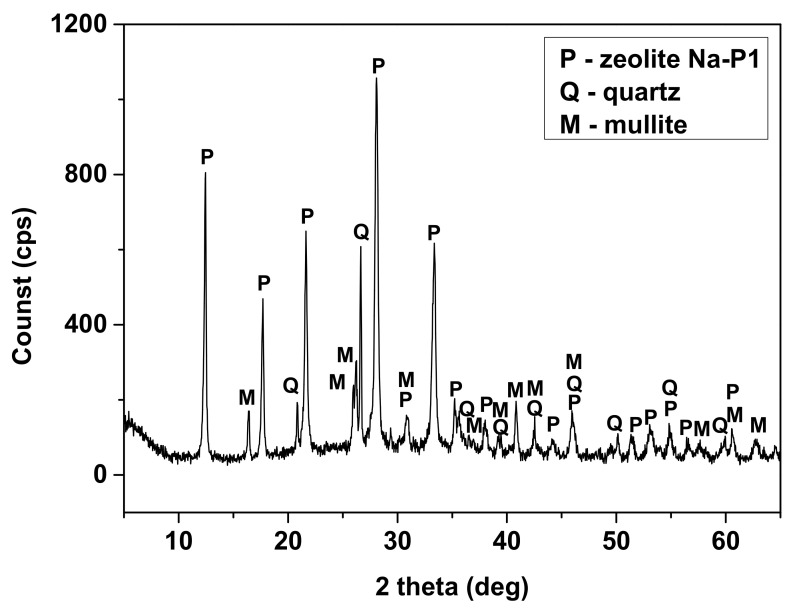
XRD patterns of NaP1.

Characteristic reflexes of NaP1 zeolite were identified (Figure 1). These were, among others: dhkl = 3.17; 7.10; 4.1; 2.68; 5.02 Å. On the diffractogram, reflexes from other phases, such as mullite (dhkl = 5.38; 3.40; 2.79; 2.59 Å) and quartz (dhkl = 4.25; 3.34; 2.45; 2.28 Å), can also be observed. Additionally, the higher background level was registered at 15–35° 2θ, which is unconverted particles of fly ash in the form of aluminosilicate glaze (so-called fly ash residue).

**Figure 2:**
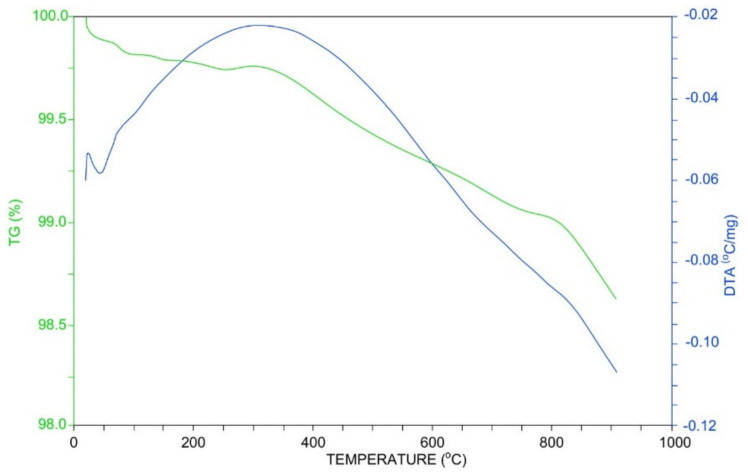
TG-DTA curves of FA.

TG-DTA analysis of the FA is presented in Figure 2. H_2_O is removed at a temperature around 373 K, Ca(OH)_2_ decomposes at 673 K and CaCO_3_ between 873 and 1023 K. A significant portion of this CaCO_3_ was not present in the fly ash originally, but is the result of a reaction between CaO (resulting from Ca(OH)_2_ decomposition) and CO_2_ [21].

**Figure 3:**
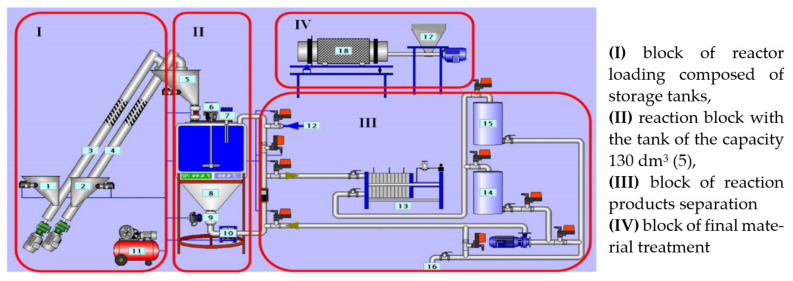
Production of zeolite NaP1.

Production of zeolite NaP1 was performed based on the hydrothermal synthesis of FA with NaOH, according to the scheme presented in Figure 3.

CS in the form of powder or flakes is characterized by low mechanical strength. Considering its pK_a_ (6.5), CS solution can be easily obtained using dilute acidic liquors. Among inorganic and organic acids, hydrochloric, phosphoric, and nitric acids; as well as formic, acetic, propionic, lactic, citric, glycolic, or succinic acids, are characterized by appropriate properties and can be used as a diluent of CS. Based on our previous results, glycolic acid was chosen, and this step significantly contributed to reduce the solution’s viscosity.

The obtained material NaP1CS is non-toxic and characterized by useful properties. The leaching procedure revealed that toxic metal ions, such as mercury, cadmium, and lead ions, are not present in the solution after 6 h treatment by H_2_O. Similar results were obtained in [22,23]. The obtained zeolite is also characterized by a high value of CEC. It is known that natural zeolites possess CEC values lower than 1.5 meq/g. For NaP1 and NaP1CS, these values were equal to 0.78 meq/g and 1.7 meq/g, respectively. It should be added that the fertilizers based on the fly ash and zeolites are not soluble in soil water and, therefore, they remain in the soil so the effect of the soil acidification does not occur [22]. As mentioned earlier, they also contain Fe(III) (7.75%), Ca(II) (1.62%), Mg(II) (1.20%), and K (0.56%) ions. Modification of NaP1 by CS is also an advantage due to the nitrogen content. The result of CHN analysis of CS and NaP1CS is presented in Table 2, showing successful modification. As for the CHN analysis, the C/N ratio increased after modification by chitosan. That fact confirms grafting of NAP1 surface by CS.

**Figure 4:**
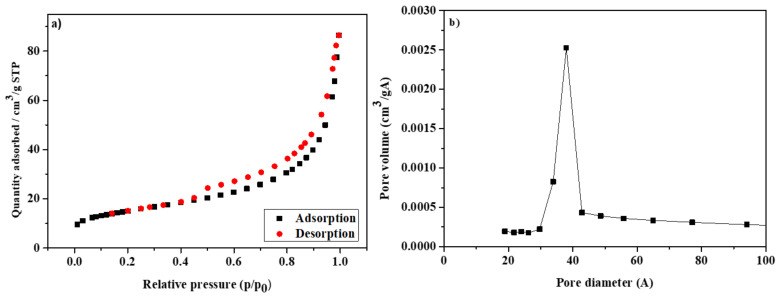
(**a**) Nitrogen adsorption–desorption isotherms at 77 K of NaP1CS, (**b**) pore diameter distribution of NaP1CS.

The nitrogen adsorption–desorption studies and pore size distribution analysis results for zeolite modified by chitosan are presented in Figure 4a,b. Currently the IUPAC defined VI types of adsorption isotherms. Consistent with the classification, the NaP1CS zeolite is characterized by the IV type of isotherm (Figure 4a) [24,25]. The same result was obtained for NaP1 (data not presented).

Table 3 presents the values of the specific surface area (S_BET_), micropore volume (V_mic_), micropore surface (S_mic_), total pore volume (V_tot_), and average pore diameter (D_av_) of NaP1CS before and after the adsorption process of Cu(II)–IDHA complexes.

According to the data in the literature [24], the values of the mesopore width are in the range 0.2–50 nm. As can be seen from Figure 4b, the pore distribution curve for NaP1CS indicates mesopores. After the adsorption process, the values of parameters characterizing the adsorbent surface slightly dropped. The total pore volume of NaP1CS was equal to 0.13 cm^3^/g, while after the adsorption process, the total pore volume achieved the value 0.11 cm^3^/g, whereas the average pore diameter after the adsorption decreased from 11 nm to 9.5 nm.

**Figure 5:**
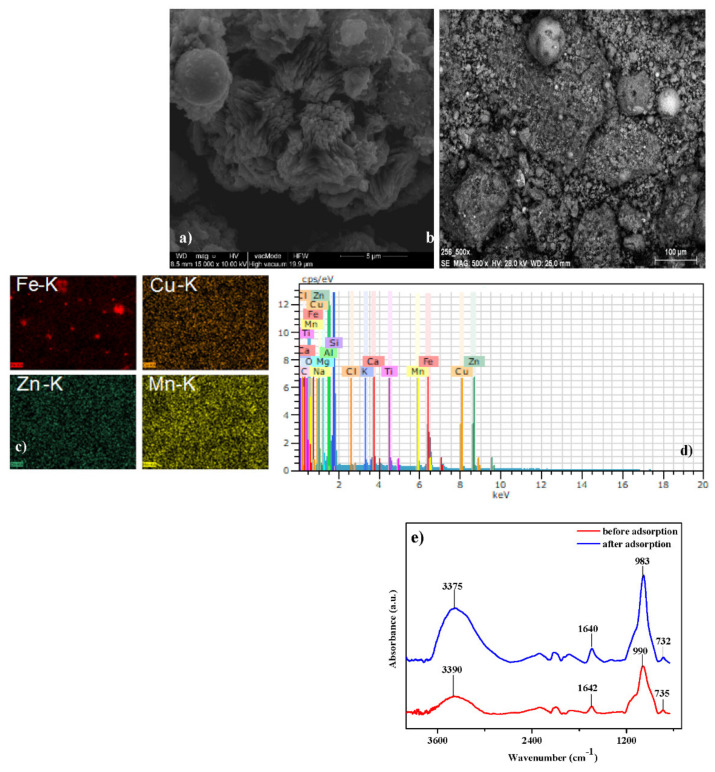
(**a**) SEM image of the NaP1, (**b**) NaP1CS after adsorption of Cu(II), Fe(III), Mn(II), and Zn(II) complexes with IDHA and corresponding X-ray maps (**c**) as well as (**d**) EDS spectra collected onto NaP1CS after adsorption of Cu(II), Fe(III), Mn(II), and Zn(II) complexes with IDHA and (**e**) FTIR spectra of NaP1CS before and after the adsorption process of Cu(II) in the presence of IDHA.

Using scanning electron microscopy combined with energy dispersive X-ray spectroscopy (SEM–EDS) [26], it was possible to map the elemental distribution within the samples before and after adsorption (Figure 5). Figure 5a,b present scanning electron microscope images of this sample before and after modification. Figure 5c displays the mapping of Fe(III), Cu(II), Zn(II), and Mn(II) after sorption process, and Figure 5d shows EDS spectra collected onto NaP1CS after the adsorption.

The chemical analysis indicated that the main elements of NaP1CS (besides O (43.75%)) were Al (17.26%) and Si (17.10%). The analysis results also showed Fe (7.75%); C (6.77%); Na (2.67%); Ca (1.62%); Mg (1.20%); and small amounts of Ti (0.96%), K (0.56%), Cu (0.26%), S (0.08%), and P (0.02%). It can be seen that, for the sample after adsorption, the spectra of Cu(II), Fe(III), Mn(II), and Zn(II) appeared. For example, the mass percentage (%) of Fe(III) and Cu(II) increased after adsorption and the values 18.94% and 0.62% were achieved, respectively, indicating that the adsorption process occurs.

Figure 5e presents the FTIR spectra of chitosan-modified zeolite NaP1CS before and after adsorption of Cu(II) complexes with IDHA. Typical vibrations for zeolite are at 990 and 735 cm^−1^, which indicates the stretching Al-O-Si and Si-O bonds, respectively. The spectra of zeolite also show bands at 1640–1642 and 3375 cm^−1^, which correspond to stretching vibrations of the Si-O, H-O, and stretching-OH attached to aluminum, respectively. The presence of -NH_2_ groups of chitosan and -OH and -NH groups is also evident. After adsorption, the intensity of the chitosan groups with -OH (3390 cm^−1^) and amide peaks (1642 cm^−1^) increases [27]. This can be due to the presence of -OH and -NH groups of the iminodisuccinic acid that interact with the chitosan groups on the adsorbent surface. Moreover, the carboxylic group present in iminodisuccinic acid is characterized by the C=O stretching vibrations, which are in the region of 1600–1700 cm^−1^ [28]. Thus, the amide peaks coincide with the C=O ones with the wavenumber at 1640 cm^−1^. Intensity of stretching Al-O-Si bond peaks also increases after adsorption. In FTIR spectra of the CS, the typical band at 3291cm^−1^ attributed to the stretching vibrations of the -OH bond is present. The band at 1591 cm^−1^ corresponds to the N-H bond of the acetyl group while 1024 cm^−1^ was attributed to the C-O-C bond [29]. In the spectrum of NaP1CS, the band at 3940 cm^−1^ corresponds to the Si-OH and Al-OH groups, as well as -OH groups.

### 3.2. Sorption Properties of NaP1CS

#### 3.2.1. Effect of pH and pH_ZPC_

**Figure 6:**
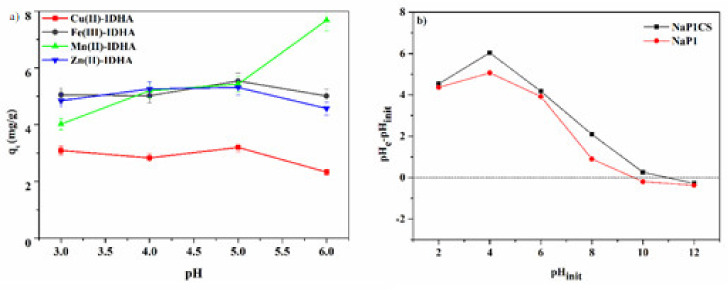
Influence of pH (**a**) on adsorption of Cu(II), Fe(III), Mn(II), and Zn(II) complexes with IDHA onto NaP1CS (m = 0.1 g, t = 120 min., A = 7, 180 rpm, pH = 3–6) and (**b**) pH_PZC_ value of NaP1Cs and NaP1.

The adsorption process is largely affected by the pH value of the solution. To determine the appropriate conditions for the adsorption process, pH values in the range from 3 to 6 were chosen. It was found that the first parameter under study that significantly affected adsorption capacity was pH (Figure 6a).

It can be seen that, for most of the complexes under study, adsorption capacity increases with increasing pH value to 5 and then starts to decrease. Only in the case of Mn(II)–IDHA =1:1 complexes can it be observed that the adsorption capacity increases with the increasing pH value above 5. Since a pH equal to 5.0 turned out to be optimal for the adsorption process, it was chosen in further experiments.

#### 3.2.2. Effect of Time—Kinetic Studies

**Figure 7:**
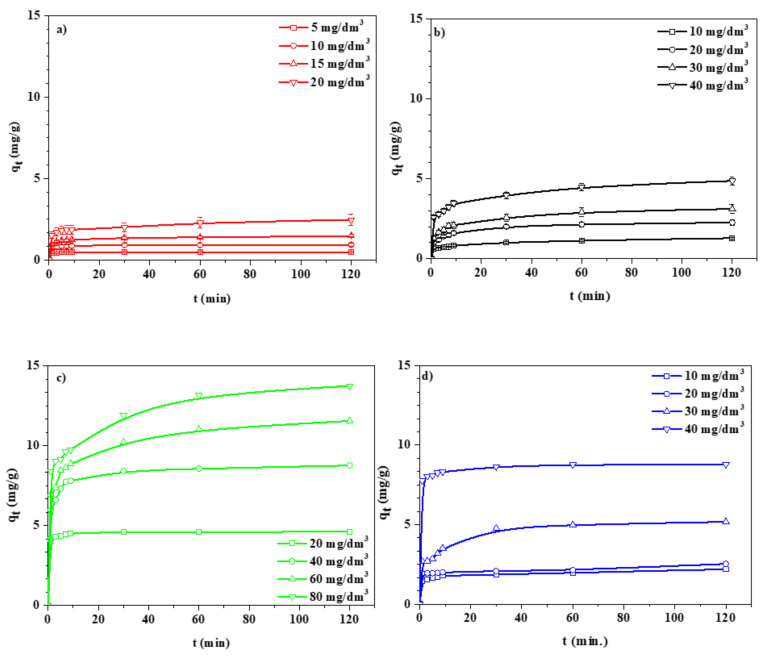
Influence of initial concentration on adsorption onto NaP1CS (**a**) Cu(II)–IDHA, (**b**) Fe(III)–IDHA, (**c**) Mn(II)–IDHA, (**d**) Zn(II)–IDHA (m = 0.1 g, t = 120 min., A = 7, 180 rpm, pH = 5).

Kinetic experiments were performed by the static (batch) method [30,31]. The effect of the initial concentration on the adsorption of Cu(II), Fe(III), Mn(II), and Zn(II) IDHA complexes onto NaP1CS is shown in Figure 7. It was found that, for the higher initial concentrations of complex solutions, higher values of adsorption capacity (q_t_) were obtained. For all examined solutions, it was proven that the maximum values of adsorption capacity were obtained for Mn(II), then for Zn(II), Fe(III), and Cu(II) complexes. Therefore, it can be assumed that studied complexes have an affinity for the surface of NaP1CS in the following order: Mn(II) > Zn(II) > Fe(III) > Cu(II). In the initial stages of adsorption, the adsorption capacity q_t_ increased rapidly. It indicates that the adsorption rate is proportional to the number of unoccupied sites on the zeolite. The equilibrium was achieved after about 5–60 min depending on the system. The kinetic parameters are presented in Table 4.

The values of adsorption capacities calculated based on the pseudo-second order kinetic model were identical with the experimental data. Additionally, the determination coefficients R^2^ were the highest for the pseudo-second order model. These data showed that the pseudo-second order kinetic model fits the best adsorption process. It was also found that the values of k_2_ decreased with the increasing initial concentration; however, the adsorption capacity increased. In accordance with the intraparticle diffusion model, the adsorption process can include three steps. In the first step, fast external surface adsorption occurs. The second step is a gradual adsorption where the intra particle diffusion rate is controlled. The third step is the final equilibrium stage, where intraparticle diffusion becomes slower [32]. According to the data presented in Table 4, it was found that the values of k_i_ increased with increasing initial complex concentration, which can be due to the greater concentration driving force [33] and can be presented as k_1_ > k_2_ > k_3_.

#### 3.2.3. Effect of Accompanied Ions

**Figure 8:**
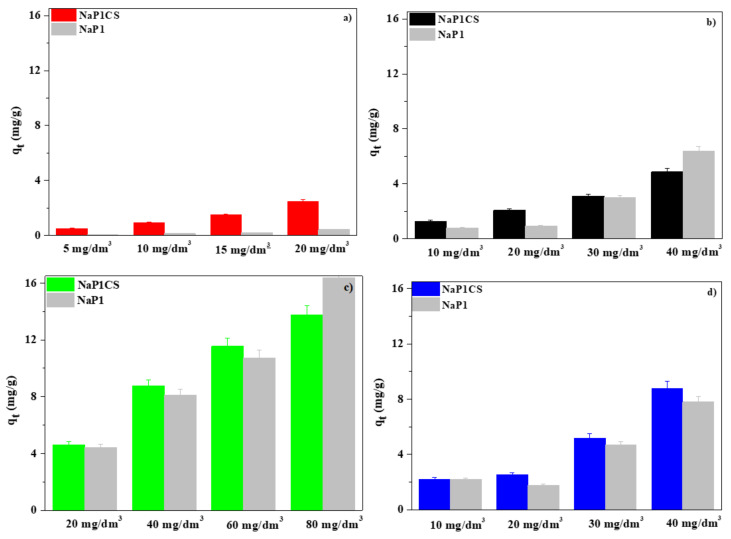
Comparison of the values of sorption capacities obtained for (**a**) Cu(II)–IDHA, (**b**) Fe(III)–IDHA, (**c**) Mn(II)–IDHA, (**d**) Zn(II)–IDHA onto NaP1 and NaP1CS (m = 0.1 g, t = 120 min, A = 7, 180 rpm, pH = 5).

Figure 8 shows the comparison of the values of the equilibrium adsorption capacity for zeolite NaP1CS and NaP1. Of four studied solutions at different initial concentrations, NaP1CS was found to be a more effective adsorbent for Cu(II), Fe(III), Mn(II), and Zn(II) complexes with IDHA at three lowest concentrations. In the case of the system containing the highest initial concentrations of the IDHA complexes, namely, 20 mg/dm^3^ Cu(II), 40 mg/dm^3^ Fe(III), 80 mg/dm^3^ Mn(II), and 40 mg/dm^3^ Zn(II), better adsorption properties were found for zeolite NaP1 before modification toward Fe(III) and Mn(II). Modification of zeolite by chitosan (NaP1CS) was the most effective for the adsorption capacity of Cu(II) complexes with IDHA, increasing almost five times compared to NaP1.

#### 3.2.4. Effect of Concentration—Adsorption Isotherms

The adsorption data were presented using four isotherm models: Langmuir, Freundlich, Dubinin–Radushkevich, and Temkin [34,35]. To maintain the ratio of Cu(II), Fe(III), Mn(II), and Zn(II) complexes with IDHA as 1:2:4:2, the initial Cu(II), Fe(III), Mn(II), and Zn(II) complex concentrations were varied from 5–60, 10–120, 20–240, and 10–120 mg/dm^3^, respectively. Table 5 displays the isotherm parameters for the complexes under investigation onto NaP1CS.

The adsorption character was determined by means of R_L_ parameter, obtained from the Langmuir model. As shown in Table 5, the R_L_ values are in the range of 0.135–0.802, which indicates that the adsorption process is favourable.

Moreover, the beneficial character of adsorption can also be determined by n values from the Freundlich model, which should be in the range 1–10 [36]. The n values are equal to 1.46, 1.57, 5.78, and 2.37 for Cu(II), Fe(III), Mn(II), and Zn(II) complexes, respectively.

The value of mean free energy of adsorption E predicts the adsorption character, and it can be calculated based on the Dubinin–Radushkevich isotherm. According to the literature data, the value of E in the range 16–40 kJ/mol or 20–40 kJ/mol indicates chemical adsorption, while values lower than 8.0 kJ/mol indicate the physical adsorption [37,38,39]. The values of E in the range 8.69–17.01, listed in Table 5, indicate that the ion exchange mechanism occurs.

Calculated on the basis of the Temkin isotherm, the highest value (2.5 kJ/mol) of adsorption heat was found for Cu(II)–IDHA = 1:1, while the equilibrium binding constant corresponding to the maximum binding energy was 1.8 dm^3^/g [40]. From all investigated isotherm models, the values of the determination coefficient R^2^ were the highest (0.94–0.99) for the Langmuir isotherm in the case of Cu(II), Mn(II), and Zn(II) complexes. It indicates that the Langmuir isotherm best fits the adsorption process. For Fe(III)–IDHA = 1:1, the maximum determination coefficient R^2^ (0.97) was obtained for the Freundlich and Dubinin–Radushkevich isotherms, whereas for the Langmuir isotherm it was lower, being 0.93.

#### 3.2.5. Comparison of Efficiency of Different Sorbents

Based on the obtained results and considering the minimum concentrations of microelements in the fertilizers with trace elements, which, according to the German Fertilizer Ordinance [41], should be equal to 0.02% for copper, 0.04% for iron, 0.02% for manganese, and 0.002% for zinc, it is evident that obtained values are even higher than those for commercial products, which should be greater than 1% for iron and 0.2% for manganese. In the paper [42], it was found that zeolite NaP1 obtained from fly ash effectively sorbed NH_4_^+^ and Zn(II) ions. However, the sorption of Mg(II), Ca(II), and K(I) ions was lower from the surface water treatment plant effluent containing 33.3 mg/dm^3^ of Ca(II), 15.9 mg/dm^3^ of K(I), 3.66 mg/dm^3^ of Mg(II), 208 mg/dm^3^ of Na(I), 0.8 mg/dm^3^ of NH_4_^+^, 0.03 mg/dm^3^ Ni(II), and 0.12 mg/dm^3^ Zn(II). For the simultaneous sorption of Ni(II) ions, further studies are needed to reduce this effect. In [43], NaP1 was used to evaluate adsorption capacities toward NH_4_^+^, Ba(II), Cd(II), Co(II), Cu(II), Ni(II), Pb(II), and Zn(II) ions. The following affinity series was established: Ba(II) > Cu(II) > Cd(II) > Zn(II) > Co(II) > Ni(II). In [44], the affinity series for alkaline and alkali earth metal ions was found: Ca(II) > Rb(I) > K(I) > Na(II) > Li(I). However, the results presented in [45] demonstrate that NaP1 is characterized by higher affinity for heavy metal ions than Ca(II) or Mg(II).

#### 3.2.6. Effect of Temperature

Table 6 presents the thermodynamic parameters for the adsorption of Cu(II), Fe(III), Mn(II), and Zn(II) complexes with IDHA onto NaP1CS. With increasing temperature (from 293 to 333 K), the adsorption capacity of NaP1CS increases compared to all examined complexes. Thus, the adsorption process is more effective at higher temperatures. As can be seen, the ΔH° values are positive, which indicates that the adsorption process is of endothermic nature [32]. In accordance with the literature data, the value of ΔH° in the range of 2.1–20.9 kJ/mol indicates physical adsorption, while chemical adsorption is characterized by a higher value of the enthalpy (80–200 kJ/mol) [46]. Thus, the ΔH° values of Cu(II), Fe(III), Mn(II), and Zn(II) complexes with IDHA adsorption onto NaP1CS indicate physical adsorption processes. The positive values of ΔS° indicate increased randomness at the solid–solution interface during adsorption of Cu(II), Fe(III), Mn(II), and Zn(II) complexes onto NaP1CS (Table 6). The negative values of ΔG° indicate that adsorption of Cu(II), Fe(III), Mn(II), and Zn(II) complexes onto NaP1CS was of a spontaneous nature. Based on the data presented in Table 6, it can be seen that the value of ΔG° increases with increasing temperature. This highlights that, at higher temperatures, the adsorption process is more energetically favorable [46].

### 3.3. Desorption Process

The desorption process was conducted to regenerate the adsorbent. The results obtained for the desorption process are presented in Table 7. Of five solutions selected for the desorption experiment, the best results were achieved using HCl. Although this acidic solution will not be applied during the release of both metal ions and their complexes in the soil solution, the desorption effectiveness indirectly indicates the sorption mechanism.

The largest recovery with HCl was obtained for Fe(III) complexes, the desorption percentage D(%) was equal to 91.4%, then, for Zn(II) 79.7%, Cu(II) 74.1%, and Mn(II) 65.4% complexes with IDHA. For Fe(III) complex desorption, all other examined solutions were ineffective. However, using a solution of NaCl, the following D(%): 25.1, 35.2, and 37.5%, were achieved for Cu(II), Zn(II), and Mn(II) complexes with IDHA, respectively. The NaOH and CH_3_OH solutions were the least effective for Cu(II) and Mn(II) complexes; the D(%) were no higher than 4.4%. In turn, for desorption of Zn(II), the least effective solution was KCl, for which the desorption percentage was equal to 0.6%. Dinu et al. found that HCl was also the most effective solution for desorption of Cu(II), Co(II), and Ni(II) obtained for the chitosan/clinoptilolite composite [30]. As well in the case of magnetic chitosan/Al_2_O_3_/iron oxide nanoparticles [35], the desorption process of Methyl Orange was performed using the HCl solution.

### 3.4. Mechanizm of Sorption of [M(idha)]^(n−m)−^ Complexes

Chitosan surface charge on the surface of NAP1CS is dependent on the pH of the solution. In acidic solutions, ready penetration of acid molecules between CS molecules and destruction of crystal structure are observed. Additionally, in an acidic medium, the amino functional groups of CS on the adsorbent surface are protonated [30]. This affects the sorption of anionic complexes of metal ions with IDHA. Metal ions in the presence of the chelating agent IDHA form the complex, which can be shown as follows [31]:

M^m+^ + idha^n−^ ⇄ [M(idha)]^(n−m)−^, (10)

**Figure 9:**
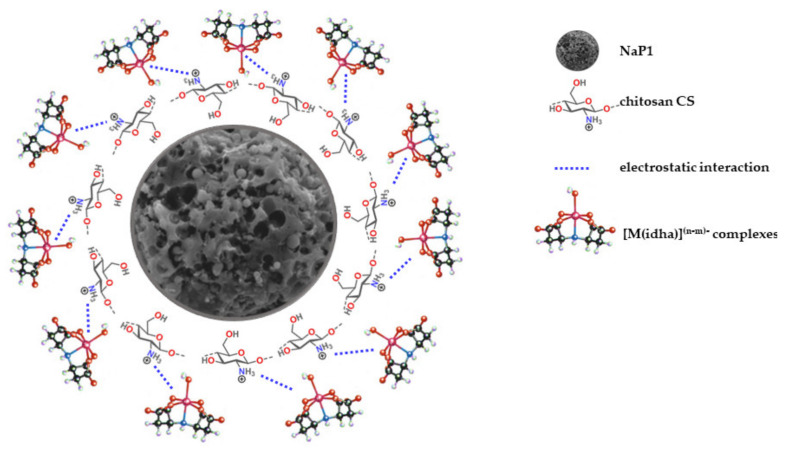
Mechanism of sorption of [M(idha)]^(n−m)−^ complexes onto NaP1CS.

The functional groups, such as hydroxyls, carbonyls, and amines, possess the positive charges to the surface due to the increased concentration of H^+^ ions, thus, increasing the capacity of anions of [M(idha)]^(n−m)−^ type. It is possible to assume that they can react with the protonated amine groups of CS by means of electrostatic attraction. This is because the working pH is below the pH_PZC_ of adsorbent material, which was equal to 10.81 (Figure 6b). Therefore, the surface of the absorbent material is positively charged, favouring the adsorption of [M(idha)]^(n−m)−^ complexes (Figure 9).

## 4. Conclusions

In this paper, synthetic zeolite NaP1 obtained from fly ash in the hydrothermal conversion process and then modified by chitosan (NaP1CS) was studied to verify its usefulness for further applications of the system to design slow-release fertilizers. Cu(II), Fe(III), Mn(II), and Zn(II) complexes with IDHA were chosen as model adsorbates because their ions are essential microelements that provide plant growth. IDHA was selected from other agents due to its good complexation of metal ions and low toxicity. Moreover, it is characterized by a higher biodegradability. It was found that NaP1CS is characterized by a high value of CEC equal to 1.7 meq/g. The result of CHN analysis of CS and NaP1CS reviled successful modification. Acidic pH is appropriate for the adsorption process and the maximum adsorption capacity was achieved at pH 5 during 1 h. The adsorption isotherms were well fit by the Langmuir equation, especially in the case of Cu(II), Mn(II), and Zn(II) complexes with IDHA. Only in the case of Fe(III)–IDHA was a higher value of the determination coefficient R^2^ obtained for the Freundlich and Dubinin–Radushkevich isotherms than for the Langmuir isotherm. The sorption process was well-fitted by the pseudo-second order equation. Thermodynamic parameters (ΔG^o^, ΔHo, and ΔSo) showed that the adsorption process is spontaneous and endothermic in nature. The increase of temperatures has a positive influence on the adsorption process. Based on desorption studies, the highest desorption percentage was achieved using the 0.5 M HCl solution. Efficiency of the desorption process amounting to 91.4% was obtained for Fe(III)–IDHA; other adsorbates were characterized by slightly lower desorption percentages equal to 79.7%, 74.1% and 65.4% for Zn(II)–IDHA, Cu(II)–IDHA, and Mn(II)–IDHA, respectively. According to the obtained experimental data, chitosan-modified zeolite NAP1CS is characterized by higher adsorption capacity values compared to NaP1. Future work will be a comparative study to test the application of NAP1CS-M(II)–IDHA composites for slow-release fertilizers.

## Figures and Tables

**Table 1:** XRF analysis of FA.

Adsorbent	Al_6_Si_2_O_13_ (%)	Fe_2_O_3_ (%)	CaO (%)	TiO_2_ (%)	SiO_2_ (%)
FA	75.80	3.50	0.00	1.80	20.00

**Table 2:** Element analysis of CS and NaP1CS.

Element	CS	NaP1CS
C (%)	40.72	10.59
N (%)	7.25	1.19
H (%]	3.91	1.46

**Table 3:** Surface characteristics of NaP1CS before and after adsorption of Cu(II)–IDHA complexes.

Parameter	Value	
	NaP1CS before Adsorption	NaP1CS after Adsorption
S_BET_ (m^2^/g)	53.5	53.4
V_mic_ ^(a)^ (cm^3^/g)	0.004	0.004
S_mic_ ^(a)^ (m^2^/g)	10.68	10.47
V_tot_ ^(b)^ (cm^3^/g)	0.13	0.11
D_av_ ^(c)^ (Å)	111	95

^(a)^ calculated from t-plot; ^(b)^ determined at p/p_o_ = 0.99; ^(c)^ Barrett, Joyner, and Halenda (BJH) model.

**Table 4:** Kinetic parameters for sorption of Cu(II), Fe(III), Mn(II), and Zn(II) complexes with IDHA onto NaP1CS.

Adsorbate	C_0_ (mg/dm^3^)	q_eq_,_exp_ (mg/g)	Kinetic Parameters		
			Pseudo-First Order		
			q_eq_ (mg/g)	k_1_ (1/min)	R^2^
**Cu(II)–IDHA = 1:1**	5	0.49	57.07	0.046	0.54
	10	0.94	7.87	0.044	0.91
	15	1.48	2.81	0.026	0.84
	20	2.47	1.25	0.023	0.95
**Fe(III)–IDHA = 1:1**	10	1.28	1.59	0.025	0.98
	20	2.06	1.01	0.081	0.98
	30	3.11	1.56	0.035	0.97
	40	4.87	2.15	0.030	0.98
**Mn(II)–IDHA = 1:1**	20	4.61	2.85	0.088	0.97
	40	8.76	1.84	0.042	0.87
	60	11.55	4.07	0.035	0.97
	80	13.74	5.05	0.026	0.96
**Zn(II)–IDHA = 1:1**	10	2.20	3.68	0.027	0.99
	20	2.53	1.79	0.037	0.92
	30	5.17	4.10	0.056	0.93
	40	8.78	1.03	0.072	0.99
			**Pseudo-second order**		
			q_eq_ (mg/g)	k_2_ (g/mg·min)	R^2^
**Cu(II)–IDHA = 1:1**	5	0.49	0.49	13.609	1.00
	10	0.94	0.94	1.683	1.00
	15	1.48	1.48	0.421	0.99
	20	2.47	2.48	0.149	0.99
**Fe(III)–IDHA = 1:1**	10	1.28	1.30	0.166	0.99
	20	2.06	2.10	0.221	0.99
	30	3.11	3.18	0.080	0.99
	40	4.87	4.94	0.056	0.99
**Mn(II)–IDHA = 1:1**	20	4.61	4.62	0.999	1.00
	40	8.76	8.80	0.109	0.99
	60	11.55	11.67	0.037	0.99
	80	13.74	13.84	0.024	0.99
**Zn(II)–IDHA = 1:1**	10	2.20	2.20	0.560	0.99
	20	2.53	2.55	0.316	0.99
	30	5.17	5.60	0.020	0.99
	40	8.78	8.80	0.295	1.00
			**Intraparticle diffusion**		
			k_i_ (mg/g min^1/2^)		
			k_1_	k_2_	k_3_
**Cu(II)–IDHA = 1:1**	5	0.49	0.038	0.029	0.001
	10	0.94	0.046	0.019	0.004
	15	1.48	0.203	0.062	0.028
	20	2.47	0.236	0.064	0.079
**Fe(III)–IDHA = 1:1**	10	1.28	0.090	0.076	0.062
	20	2.06	0.312	0.158	0.009
	30	3.11	0.445	0.173	0.062
	40	4.87	0.431	0.221	0.115
**Mn(II)–IDHA = 1:1**	20	4.61	0.221	0.180	0.010
	40	8.76	0.990	0.250	0.062
	60	11.55	1.161	0.480	0.163
	80	13.74	0.877	0.800	0.338
**Zn(II)–IDHA = 1:1**	10	2.20	0.005	0.070	0.025
	20	2.53	0.215	0.039	0.020
	30	5.17	0.128	1.058	0.071
	40	8.78	0.270	0.158	0.022

**Table 5:** Isotherm parameters for Cu(II), Fe(III), Mn(II), and Zn(II) complexes with IDHA onto NaP1CS.

Adsorbate	Isotherm	Parameters			
	Langmuir	q_m_ (mg/g)	K_L_ (L/mg)	R^2^	R_L_ (-)
Cu(II)–IDHA	-	3.93	0.074	0.94	0.725
Fe(III)–IDHA		17.26	0.032	0.93	0.802
Mn(II)–IDHA		11.59	0.314	0.99	0.135
Zn(II)–IDHA		6.14	0.069	0.96	0.545
	Freundlich	K_F_ (mg/g)	n (-)	R^2^	-
Cu(II)–IDHA	-	3.53	1.46	0.85	-
Fe(III)–IDHA		1.06	1.57	0.97	
Mn(II)–IDHA		5.87	5.78	0.72	
Zn(II)–IDHA		1.11	2.37	0.80	
	Dubinin-Radushkevich	X_m_ (mg/g)	β (mol^2^/kJ^2^)	R^2^	E (kJ/mol)
Cu(II)–IDHA	-	2014.4	0.0066	0.87	8.69
Fe(III)–IDHA		964.2	0.0058	0.97	9.28
Mn(II)–IDHA		3109.9	0.0017	0.81	17.01
Zn(II)–IDHA		3476.7	0.0043	0.82	10.77
	Temkin	A (L/g)	b (kJ/mol)	R^2^	B (J/mol)
Cu(II)–IDHA	-	1.805	2.530	0.93	0.979
Fe(III)–IDHA	-	1.569	0.895	0.90	2.767
Mn(II)–IDHA	-	127.41	1.836	0.70	1.349
Zn(II)–IDHA	-	1.268	1.923	0.86	1.288

**Table 6:** Thermodynamic parameters for sorption of Cu(II), Fe(III), Mn(II), and Zn(II) complexes with IDHA onto NaP1CS.

Adsorbent	Adsorbate	(ΔH°) (kJ/mol)	(ΔS°) (J/mol K)	(ΔG°) (kJ/mol)		
				293 K	313 K	333 K
NaP1CS	Cu(II)–IDHA	5.94	16.6	−10.33	−11.74	−12.53
	Fe(III)–IDHA	16.80	49.1	−14.41	−16.55	−18.67
	Mn(II)–IDHA	19.70	44.4	−10.02	−12.42	−14.05
	Zn(II)–IDHA	3.91	3.8	−11.82	−12.87	−13.97

**Table 7:** Percentage desorption DP(%) of Cu(II), Fe(III), Mn(II), and Zn(II) complexes with IDHA for NaP1CS.

Solution	Percentage Desorption (%)			
	Cu(II)–IDHA	Fe(III)–IDHA	Mn(II)–IDHA	Zn(II)–IDHA
0.5 M NaCl	25.1	0	37.5	35.2
0.5 M NaOH	1.3	0	0.3	25.7
0.5 M KCl	24.6	0	26.9	0.6
0.5 M CH_3_OH	4.4	0	2.7	14.2
0.5 M HCl	74.1	91.4	65.4	79.7

## Data Availability

The data presented in this study are available on request from the corresponding author.
